# Beyond the T-junction: Reinforce strategic sites in superomedial pedicle mammoplasties using a dual dermal flap technique

**DOI:** 10.1016/j.jpra.2025.09.008

**Published:** 2025-09-20

**Authors:** Clelia Dogny, Matteo Scampa, Tom Membrez, Giulia Frigerio, Patricia E. Engels, Daniel Felix Kalbermatten, Dominik André-Levigne

**Affiliations:** a Department of Plastic, Reconstructive & Aesthetic Surgery, University Hospitals of Geneva, Rue Gabrielle-Perret-Gentil 4, Geneva, Switzerland; b Department Diagnostique, Pathologies Clinique, University Hospitals of Geneva, Rue Gabrielle-Perret-Gentil 4, Geneva, Switzerland

**Keywords:** Breast reduction, Dermal flap, Reduction mammoplasty, Superomedial pedicle, Superior pedicle, Wound dehiscence

## Abstract

**Introduction:**

Breast reduction using superior or superomedial pedicles provides favorable long-term aesthetic outcomes, particularly regarding breast shape and projection. However, wound dehiscence remains a concern at the T-junction and the vertical–areolar junction, with reported rates ranging from 4 % to 20 %, contributing to a higher postoperative burden, suboptimal aesthetic results, and increased healthcare costs. The development of preventive techniques is a key aspect as scar quality is considered one of the most important factors by patients. Only a few techniques in the literature describe using dermal flaps at high-tension sites to reduce tension and provide additional support and blood supply.

**Materials and Methods:**

**:** A literature review was performed via PubMed using the terms “dermal flap,” “dermo-glandular flaps,” “de-epithelialized flaps,” “breast reduction,” “mammoplasty,” “scar dehiscence,” and “scar complication.” We then present a modification of the superomedial pedicle technique that includes two small semi-circular de-epithelialized dermal flaps placed at the T-junction and the vertical–areolar junction. These aim to offload tension and reinforce vascular support at high-risk sites.

**Results:**

Three studies described dermal flap use at the T-junction, reporting a reduction in scar-related complications from 4.3 % to 1.4–2.9 %. However, none addressed the vertical–areolar junction. Our technique is, to our knowledge, the first to reinforce both areas simultaneously.

**Conclusion:**

We propose a simple modification to the standard technique using small dermal flaps at strategic points to reinforce T-inverted scars for superomedial and superior pedicles. Building on previous studies, This approach aims to enhance healing and reduce complications without increasing operative time.

## Introduction

Breast hypertrophy is a prevalent condition that can significantly impact patients’ quality of life, leading to musculoskeletal pain such as neck, shoulder, and back pain, limited arm mobility due to mechanical impingement, skin fold maceration, increased thoracic kyphosis, and psychological distress. Breast reduction surgery has been shown to greatly enhance the quality of life of women suffering from symptomatic breast hypertrophy.^1^, ^2^, ^3^ Breast reduction is a widely performed procedure, with 76,000 surgeries conducted in the U.S. in 2023.^4^ This surgery must be both functional and aesthetic, ensuring long-lasting and satisfactory results while minimizing complications. Various skin incision patterns and dermoglandular pedicle choices are described, each offering distinct advantages and limitations. In Europe, the superomedial pedicle is widely used as an alternative to the inferior pedicle. The superomedial pedicle has been reported to offer a more aesthetically pleasing long-term outcome, with less bottoming-out, a more natural breast shape, and better preservation of NAC sensitivity, while maintaining similar to more favorable reported complication rates.^5^, ^6^, ^7^, ^8^

One of the most common complications of breast reduction is wound dehiscence, particularly at the two areas where incisions cross, i.e. at the T-junction between the vertical and horizontal suture and at the junction between the vertical and periareolar suture. In cases where superomedial or superior pedicles are used, dehiscence exposes the underlying adipose and glandular tissue often requiring multiple dressings before wound closure.^6^^,^^9^, ^10^, ^11^ Rates of wound dehiscence in breast reduction surgery have been reported to range from 4 % to 20 % leading to increased postoperative visits, potentially less favorable aesthetic outcomes and increased costs.^9^^,^^11^, ^12^, ^13^, ^14^, ^15^ The development of techniques to prevent dehiscence is a key aspect of breast reduction surgery as the scar quality is considered one of the most important factors by patients.^12^^,^^16^, ^17^, ^18^ The T-junction and the junction of the vertical and periareolar scar are critical weak points.^9^^,^^15^ Only a few techniques in the literature describe the use of dermal flaps at strategic scar sites to reduce tension and provide additional support in high-risk areas, aiming to improve wound healing and minimize complications.^18^, ^19^, ^20^

In this article, we aim at providing a comprehensive overview of dermal flap techniques to prevent wound dehiscence in mammoplasty previously described in the literature. We present our modified superomedial pedicle technique incorporating two small, semi-circular de-epithelialized dermal flaps positioned at strategic high-risk points for wound complications.

## Materials and methods

### Literature review

A literature review on the use of small dermal flaps strategically placed to reduce tension and prevent wound dehiscence in reduction mammoplasty was performed. A PubMed search query was conducted using the keywords: “dermal flap” “dermoglandular flaps,” “de-epithelialized flaps,” “breast reduction,” “mammoplasty,” “scar dehiscence,” and “scar complication.”

Only articles specifically investigating dermal flaps and their impact on the healing process in breast reduction were included. All pedicle types were included except for the inferior pedicle, as it already features dermal interface of the inferior pedicle below the wise pattern scar.

## Results

Three articles investigating the use of small dermal flaps to enhance wound healing and minimize scar-related complications in key areas were identified.^18^, ^19^, ^20^

**Figure 1:**
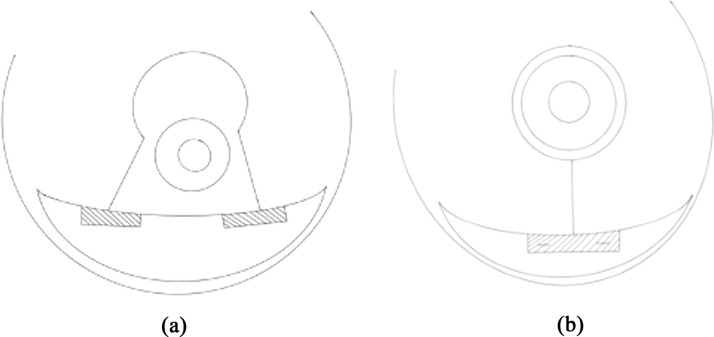
Simplified technique of the Plaza et al. technique.^18^

De la Plaza et al. described the use of two dermal flaps at the T-junction in a wise pattern supero-medial pedicle breast reduction.^18^ They designed two 5 cm long rectangular de-epithelialized dermal flaps, positioned at the edges of the skin flaps created by the vertical and horizontal incisions (Figure 1). These flaps were overlapped and sutured together, one over the other, at the level of the T-zone, and then secured to the musculo-aponeurotic wall. This study was a retrospective single arm study involving 136 patients and reported a 1.4 % incidence of cutaneous epidermolysis, without cases of proper wound dehiscence.

**Figure 2:**
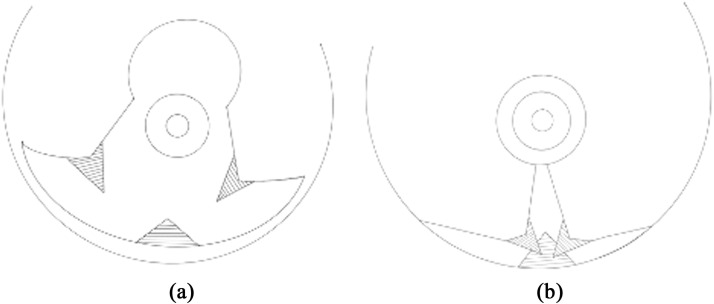
Simplified technique of the Domergue et al. technique.^20^

Domergue et al. described a technique using three dermal flaps positioned at the T-junction, arranged in a superimposed configuration for a supero-medial pedicle and wise pattern^20^ The two superior equilateral triangles measured 1.5 cm per side, and the inferior isosceles triangle had a 5 cm base and 4 cm sides (Figure 2). This study was a comparative single-center, prospective, randomized study conducted on 50 female patients. They reported a statistically significant difference, with a 2 % incidence of T-zone scar complications in the dermal flap group, compared to 16 % in the control group, and a significantly shorter healing time (19.7 vs. 25.48 days).

**Figure 3:**
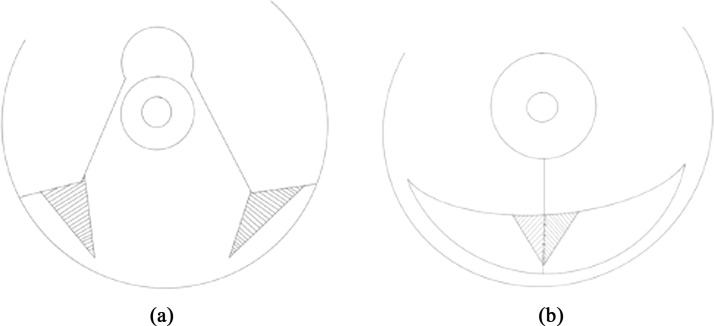
Simplified drawing of the Kalil et al. technique.^19^

Khalil et al. described the use of two triangular lipo-dermal flaps in a superomedial pedicles wise pattern reduction mammaplasty (Figure 3).^19^ These two 2 mm wide dermal flaps were created at the upper border of the horizontal incision, on either side of the vertical incision, then sutured together, forming a larger triangular structure, which was anchored to the musculo-aponeurotic connective tissue of the inframammary fold, with the final skin closure performed above it. This study was single center, single arm retrospective conducted on 173 breasts and reported a 2.9 % dehiscence rate.

### Our technique

#### Preoperative markings

The mid-axis of the thorax, then the breast and inframammary folds are marked with the patient standing. The new position of the areola is then determined following Pitanguy’s technique (projection of the infra-mammary fold on the anterior breast parenchyma).^21^ A Wise Pattern is drawn with the lateral pillars defined according to Biesenberger. Dermoglandular dog ear resection markings are adapted depending on the patient’s anatomy and tissue excess. The future superomedial NAC flap is marked with its width adapted to the desired reduction volume, but at least 8 cm to avoid vascular compromise of the NAC.

**Figure 4:**
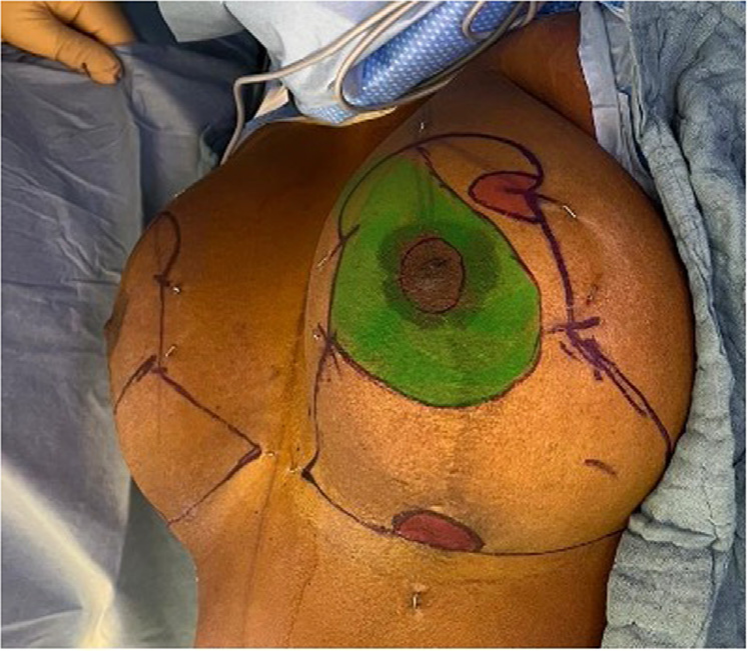
Preoperative markings.

**Figure 5:**
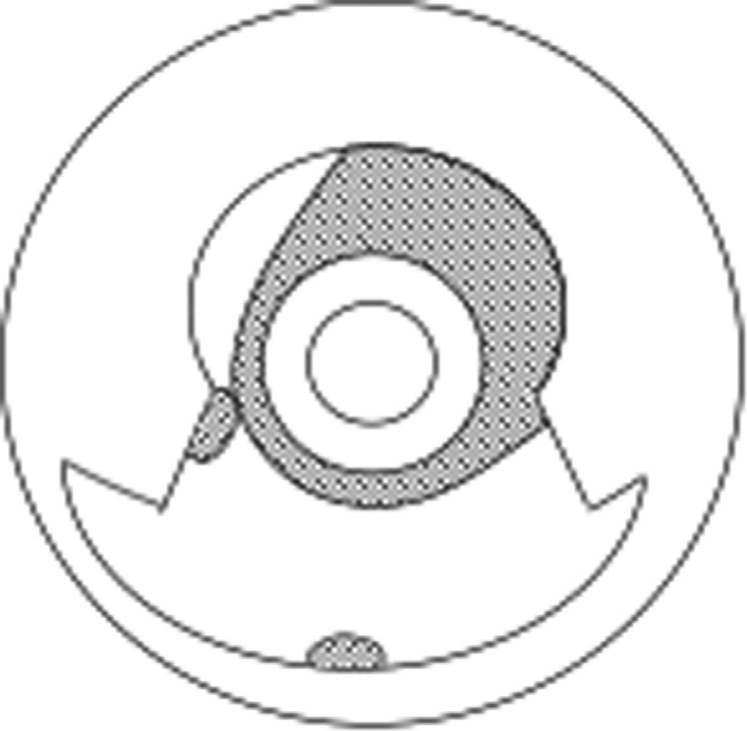
Preoperative markings, simplified version.

Two semi-circular dermal flaps (generally 2 × 2 cm but can be adapted to the breast size) are designed: one at the level of the T-junction in the inframammary fold and the second one at the superior extremity of the lateral vertical limb (Figures 4 and 5).

### Surgical technique

**Figure 6:**
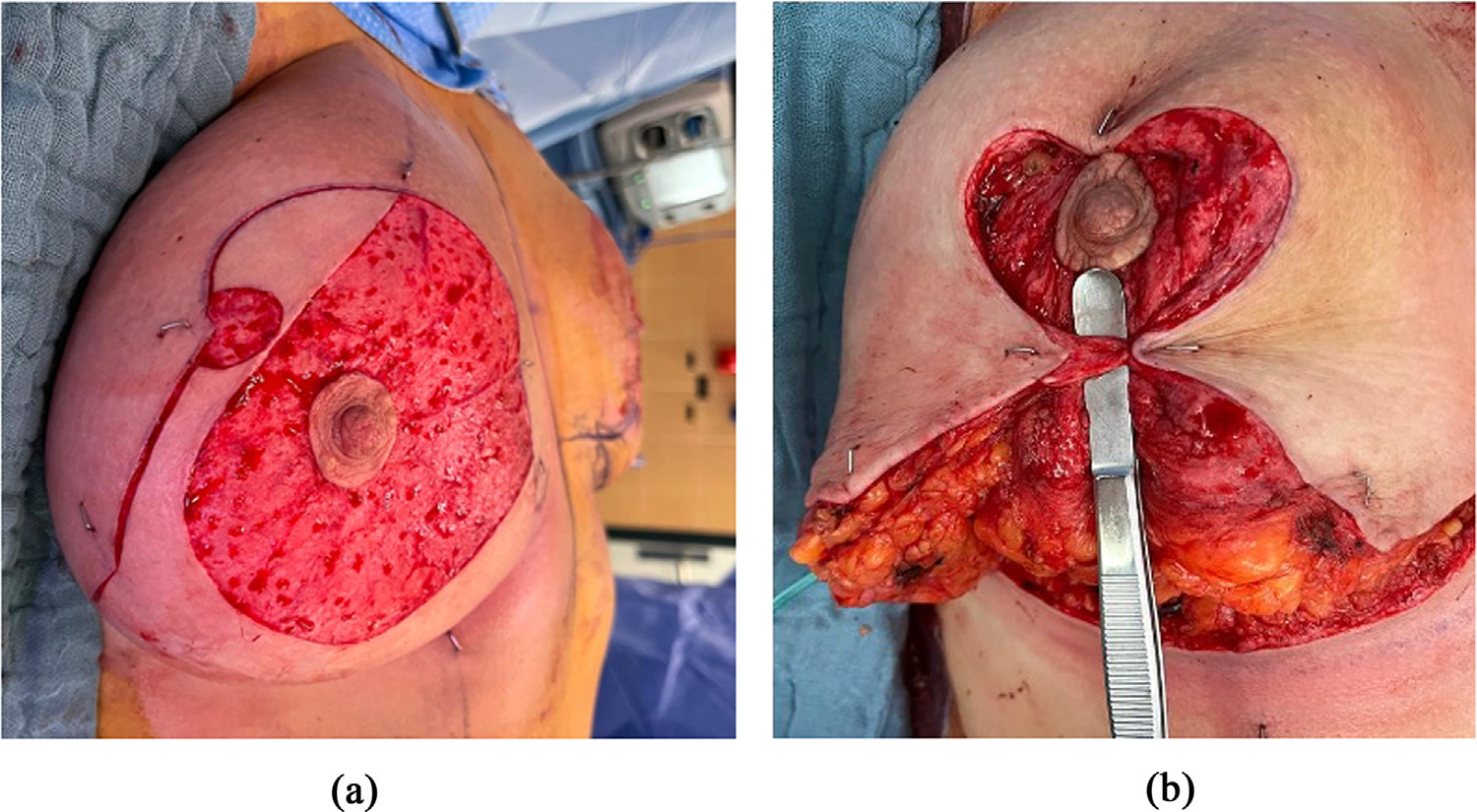
Flap in the supero-external extremity of the vertical incision.

**Figure 7:**
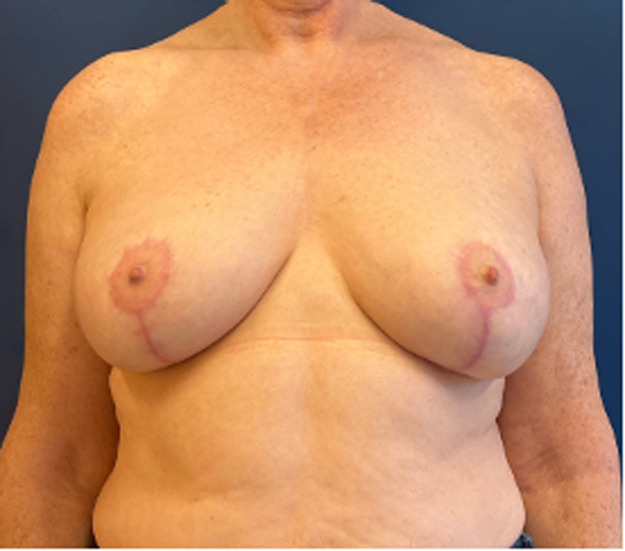
Post-operative results.

**Figure 8:**
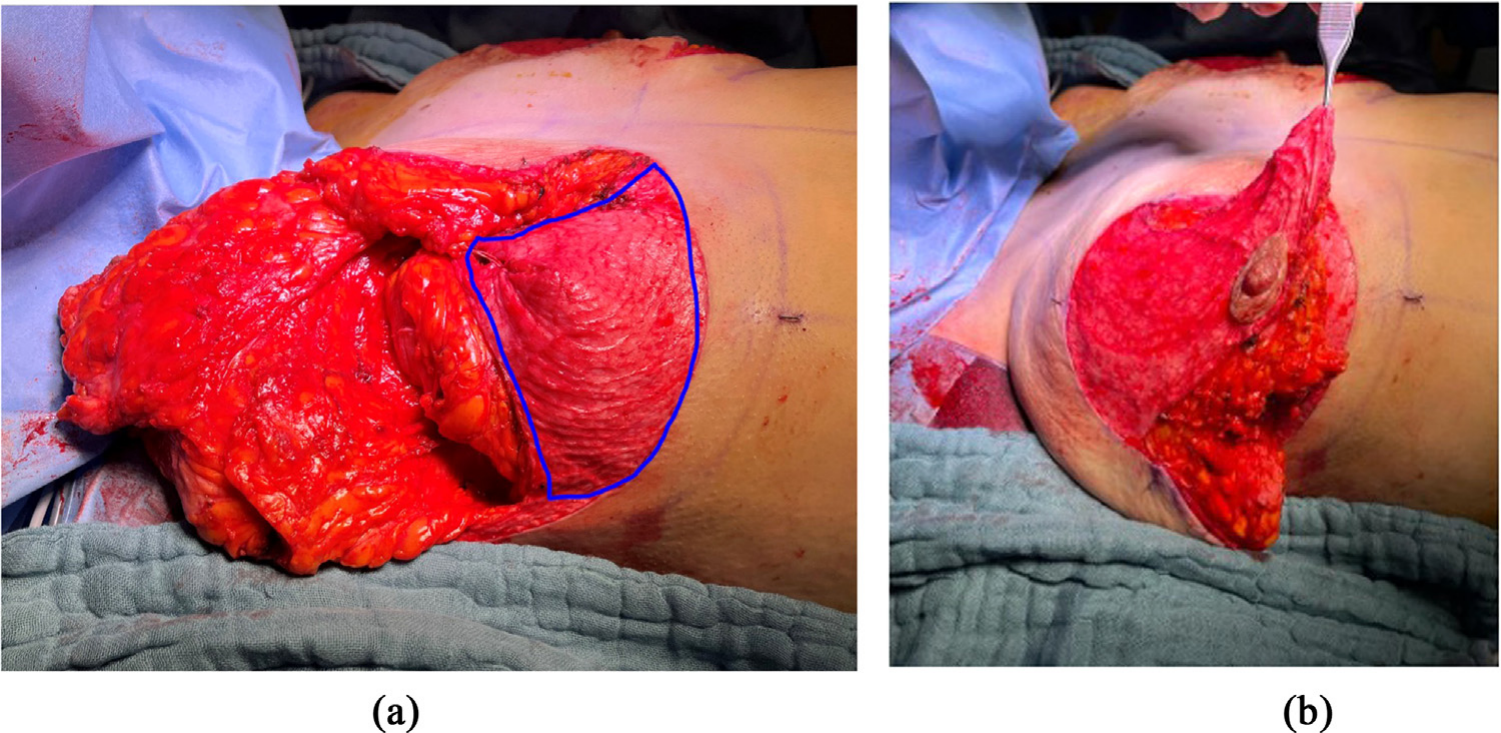
Enlarged inferior dermal flap for mastopexy.

De-epithelialization of the supero-medial pedicle and of the two previously designed dermal flaps is performed (Figures 2 and 3). The superomedial flap is then dissected to the fascia of the pectoralis muscle keeping the deep vascularization, by preserving Würinger septum.^22^ Adipose and glandular tissues of the supero-external and inferior breast are excised as well as the dermo-glandular dog ears. The two de-epithelialized flaps are positioned underneath the strategic points of the “T” junction and at the junction of the vertical incision below the areola and sutured with Monocryl 3–0 to the opposing subcutaneous tissue (Figure 6). We do not perform anchor points to the underlying pectoral muscle, as described by de la Plaza et al.,^18^ due to the risk of breast animation deformity. In mastopexy patients the inferior dermal flap can be designed much larger and positioned underneath the NAC-bearing flap in order to increase breast projection (see Figure 8). Also in these cases we recommend refraining from sutures to the muscle fascia to avoid animation. Classic layered closure with resorbable monofilament is performed. The post-operative results are shown in Figure 7.

## Discussion

In mammoplasty, the T-junction and the junction between the areola and the vertical scar are high-tension zones prone to wound dehiscence.

A few techniques to minimize wound dehiscence at the horizontal T-junction are described in the literature. Our literature review found no technique specifically addressing the junction of the vertical suture and the areola. We propose the use of small dermal flaps at both the T-junction and the junction of the vertical periareolar suture as a simple and effective method to reduce wound dehiscence and improve scar quality in mammoplasty. By redistributing mechanical tension onto the dermal flaps, these structures serve as both vascular and mechanical reinforcements, reducing stress on the overlying cutaneous sutures, therefore promoting optimized wound healing. In addition to reducing tension, dermal flaps function as anchors by providing structural supports, while also serving as a well-vascularized scaffold through the de-epithelialized flap.

We propose their use at two key locations: the T-junction and the junction of the vertical scar and the areola. While the placement of dermal flaps at the T-junction has been previously described by the aforementioned authors, to the best of our knowledge, this is the first report to describe a dermal flap specifically positioned at the junction of the vertical incision and the areola. We strongly believe this zone is subject to high tension and prone to delayed wound healing. This innovation distinguishes our method from previous techniques, which focused solely on the T-junction or used multiple overlapping flaps at the lower pole. Dermal flaps, as mentioned in the studies above provide encouraging results, with lower rates of scar-related complications compared to standard techniques (approximately 4.3 % vs. 1.4–2.9 % with dermal flaps).^12^ However, the interpretation of these findings remains limited due to the study designs with small sample sizes, absence of matched control groups and heterogeneity of the population studied.

High risk factors for poor wound healing such as smoking, high body mass index, resection weight, and operation time, are well established.^12^^,^^17^^,^^23^ For such patients, integrating the described dermal flaps is particularly relevant, as they provide an additional layer of tissue between the skin and glandular tissues as superomedian pedicles are increasingly recognized in the literature for their superior long-term outcomes, particularly in preserving breast shape and projection over time.^5^^,^^24^

The size and width of the flap at the T-junction can be adapted and be used to increase breast projection and improve shape in mastopexy. We find a large flap design particularly useful in massive weight loss patients where the flap is elevated and positioned beneath the superior or superomedial NAC-bearing flap, to enhance breast projection (see Figure 8). Several techniques associating an inferior dermoglandular flap with a superior or superomedial NAC-bearing flap have been reported to provide additional glandular support, better breast shape, projection, and long-term ptosis.^25^^,^^26^ Some authors advocate anchoring these flaps to the muscle fascia to enhance breast stability, although care should be taken to avoid breast animation.

## Conclusion

Only a few articles were found in the literature describing techniques to prevent wound dehiscence in mammoplasty. We present a dermal flap technique designed to reinforce weak points based on the concept of tension redistribution and vascular enhancement. Our technique represents an adaptation of previously described techniques by utilizing a single dermal flap at the T-junction—rather than overlapping or multiple flaps and by adding a dermal flap previously not described at the junction of the vertical periareolar suture. We believe that this minor modification can help stabilize scars without increasing operative time, ultimately promoting better postoperative healing.

## Funding

None.

## Ethical approval

Not applicable.

## Declaration of competing interest

The authors declare no conflicts of interest and received no funding for this work. Written informed consent was obtained from all patients for publication of their data/images.
